# Supplementary material for: Heterogeneity in the Drosophila gustatory receptor complexes that detect aversive compounds
Source: Nat Commun. 2017 Nov 14;8:1484. doi: 10.1038/s41467-017-01639-5 (PMC5684318; doi:10.1038/s41467-017-01639-5)
Supplement: Supplementary file 1 — Supplementary Information [file 41467_2017_1639_MOESM1_ESM.pdf]

Supplementary Figure 1

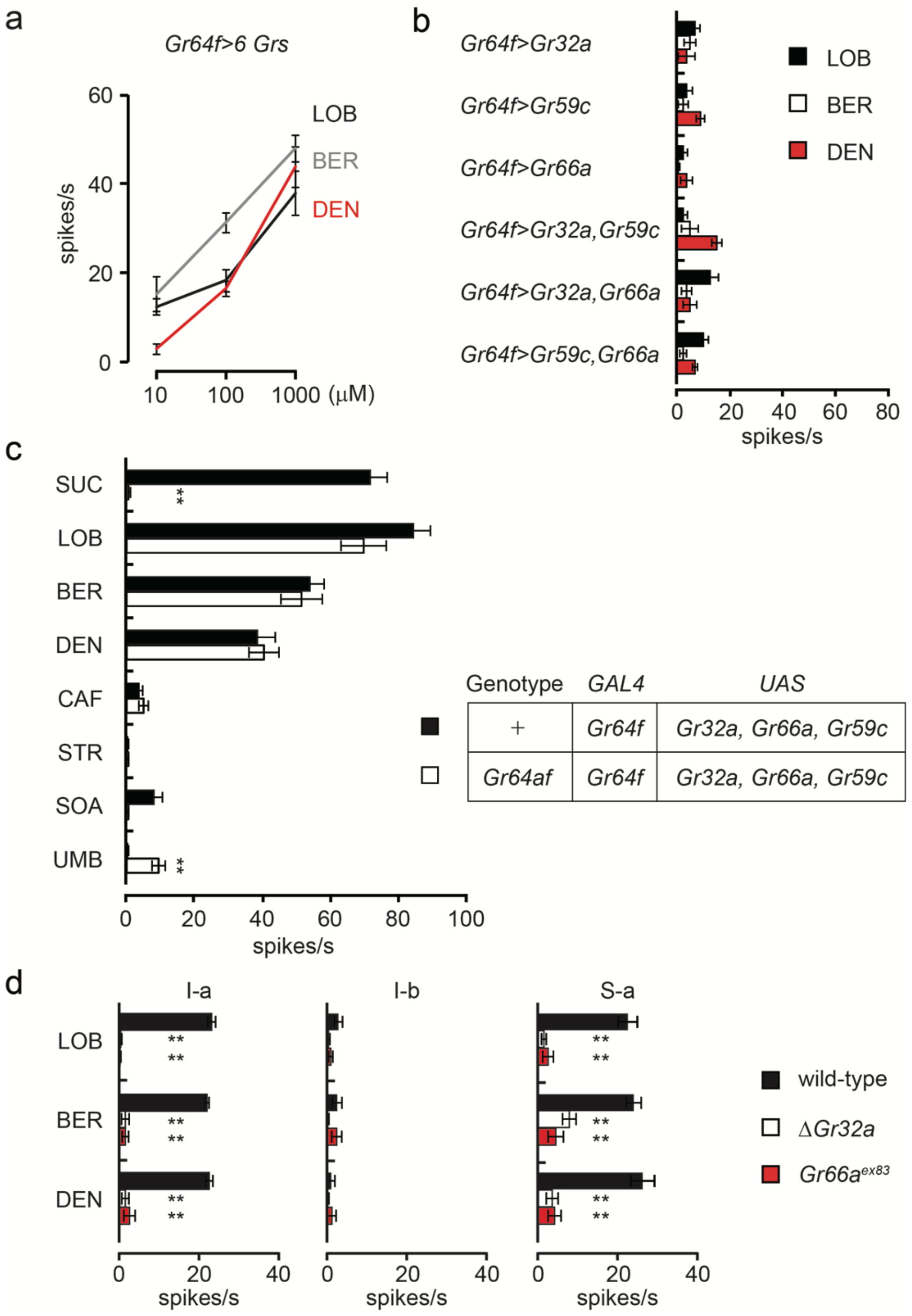

Supplementary Figure 1. The combination of *Gr32a, Gr59c*, and *Gr66a* represents the minimum requirement for ectopic responses to LOB, BER, and DEN in the sweet GRNs.

(**a**) Dose-dependent responses of the sweet GRNs expressing six bitter *Gr*s in L-type sensilla.

n = 7–11. (**b**) Mean responses elicited by 1 mM stimulation of LOB, BER, and DEN in L-type sensilla of indicated genotypes. n = 6–10. (**c**) The mean responses of *Gr64* cluster missing L-type sweet GRNs expressing *Gr32a*, *Gr59c*, and *Gr66a* induced by the indicated chemicals: 100 mM SUC, 1 mM LOB, 1 mM BER, 1 mM DEN, 5 mM CAF, 1 mM STR, 1 mM SOA, and 1 mM UMB. n = 7–13. Note that the wild-type L-type sensilla data are the same as shown in Fig. 1f. $^{**}p < 0.01$. (**d**) Mean responses elicited by 1 mM stimulation of LOB, BER, and DEN in I-a, I-b, and S-a sensilla of wild-type (black), *ΔGr32a* (white), and $Gr66a^{ex83}$ (red) flies. ANOVA followed by Tukey *post-hoc* tests or Kruskal-Wallis test followed by Mann-Whitney U *post-hoc* tests as appropriate. n = 6–15. $^{**}p < 0.01$ indicating statistical difference compared with wild-type. All data are presented as means ± S.E.M. Complete genotypes are listed in Supplementary Table 6.

Supplementary Figure 2

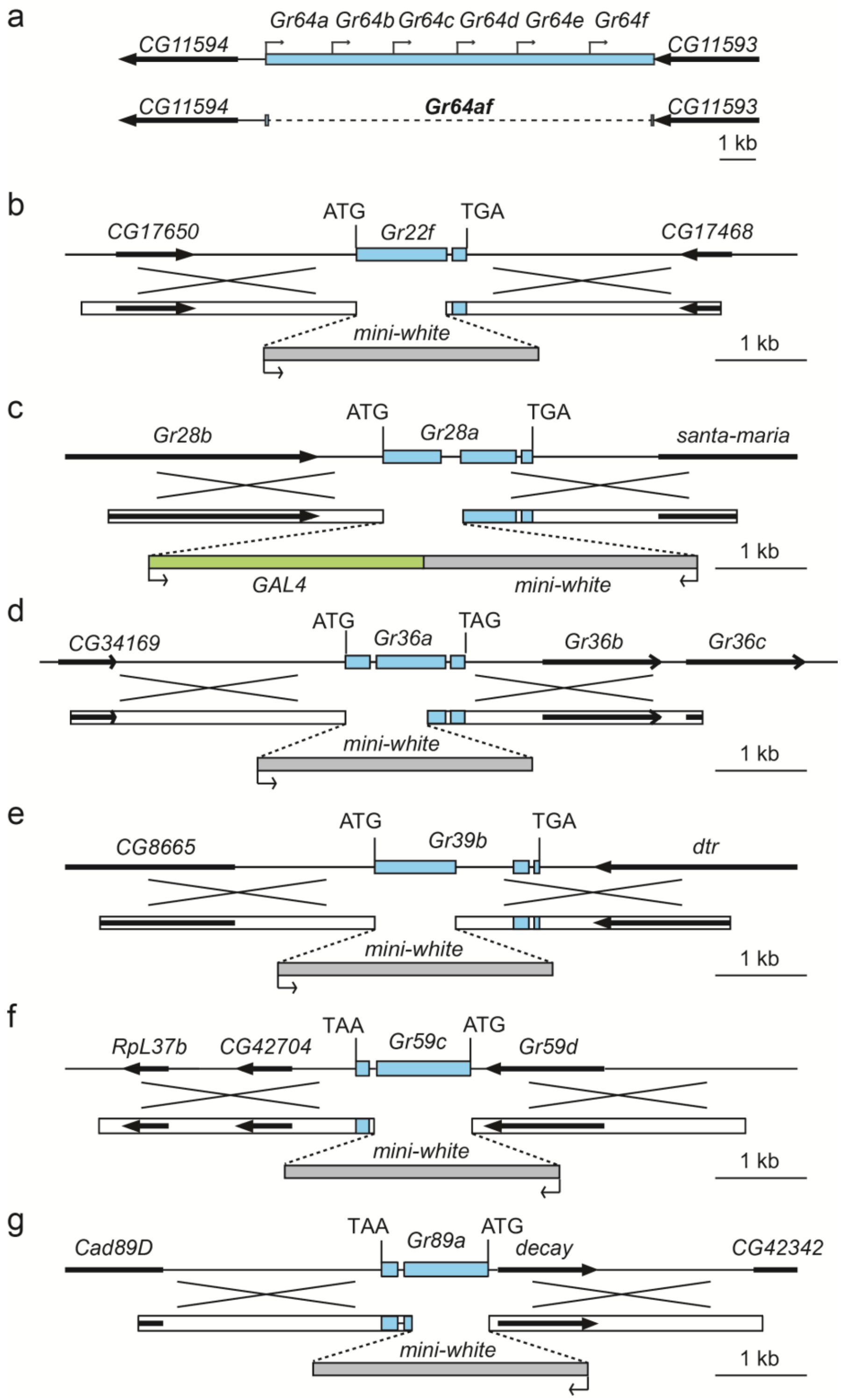

Supplementary Figure 2. Mutant generation schematics. Schematics showing the mutant generation strategies for (**a**) *Gr64af*, (**b**) *Gr22f*[1], (**c**) *Gr28a*[1], (**d**) *Gr36a*[1], (**e**) *Gr39b*[1], (**f**) *Gr59c*[1], and (**g**) *Gr89a*[1].

Supplementary Figure 3

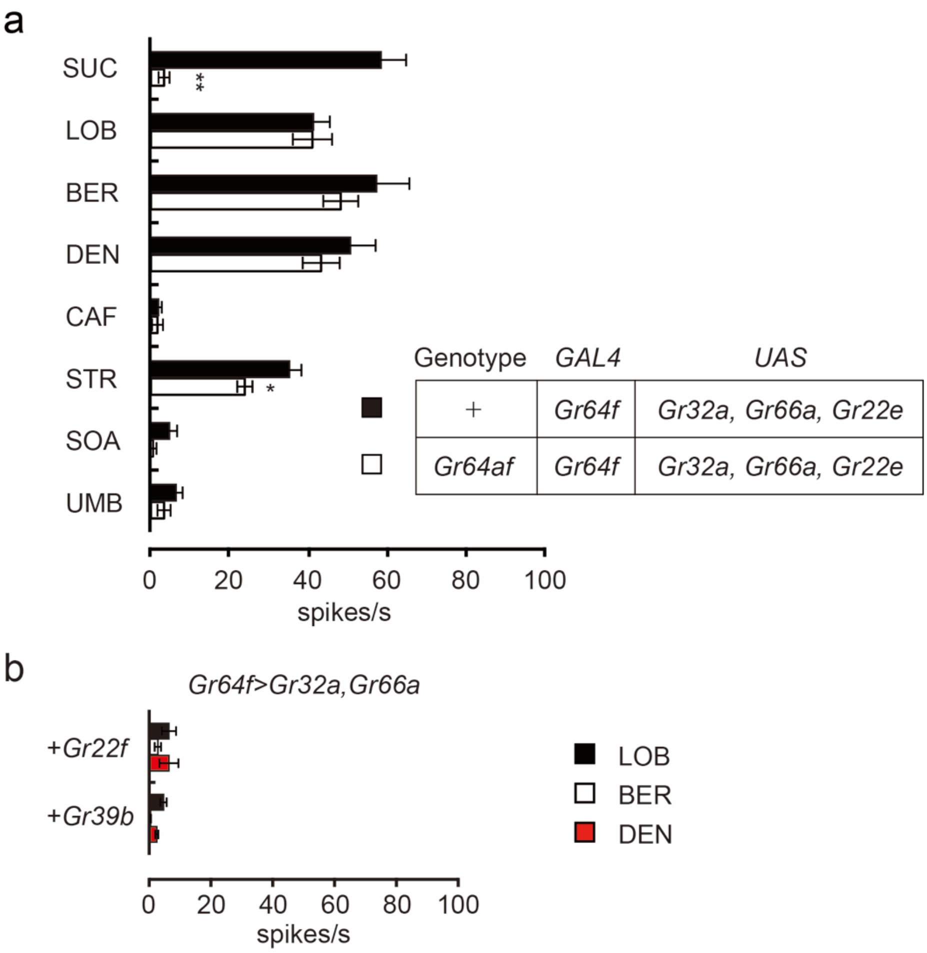

Supplementary Figure 3. The combination of *Gr22e, Gr32a,* and *Gr66a* represents the minimum requirement for ectopic responses to LOB, BER, DEN, and STR in the sweet GRNs. (**a**) The mean response of *Gr64* cluster deletion mutant L-type sweet GRNs expressing *Gr32a*, *Gr22e*, and *Gr66a* induced by the indicated chemicals: 100 mM SUC, 1 mM LOB, 1 mM BER, 1 mM DEN, 5 mM CAF, 1 mM STR, 1 mM SOA, and 1 mM UMB. n = 7–13. Note that the wild-type L-type sensilla data are the same as those shown in Fig. 3f. *p < 0.05, **p < 0.01. (**b**) Mean responses elicited by 1 mM stimulation of LOB, BER, and DEN in L-type sweet GRNs expressing either *Gr22f* or *Gr39b* in combination with *Gr32a* and *Gr66a* using *Gr64f-GAL4*. All data are presented as means ± S.E.M. Complete genotypes are listed in Supplementary Table 6.

Supplementary Figure 4

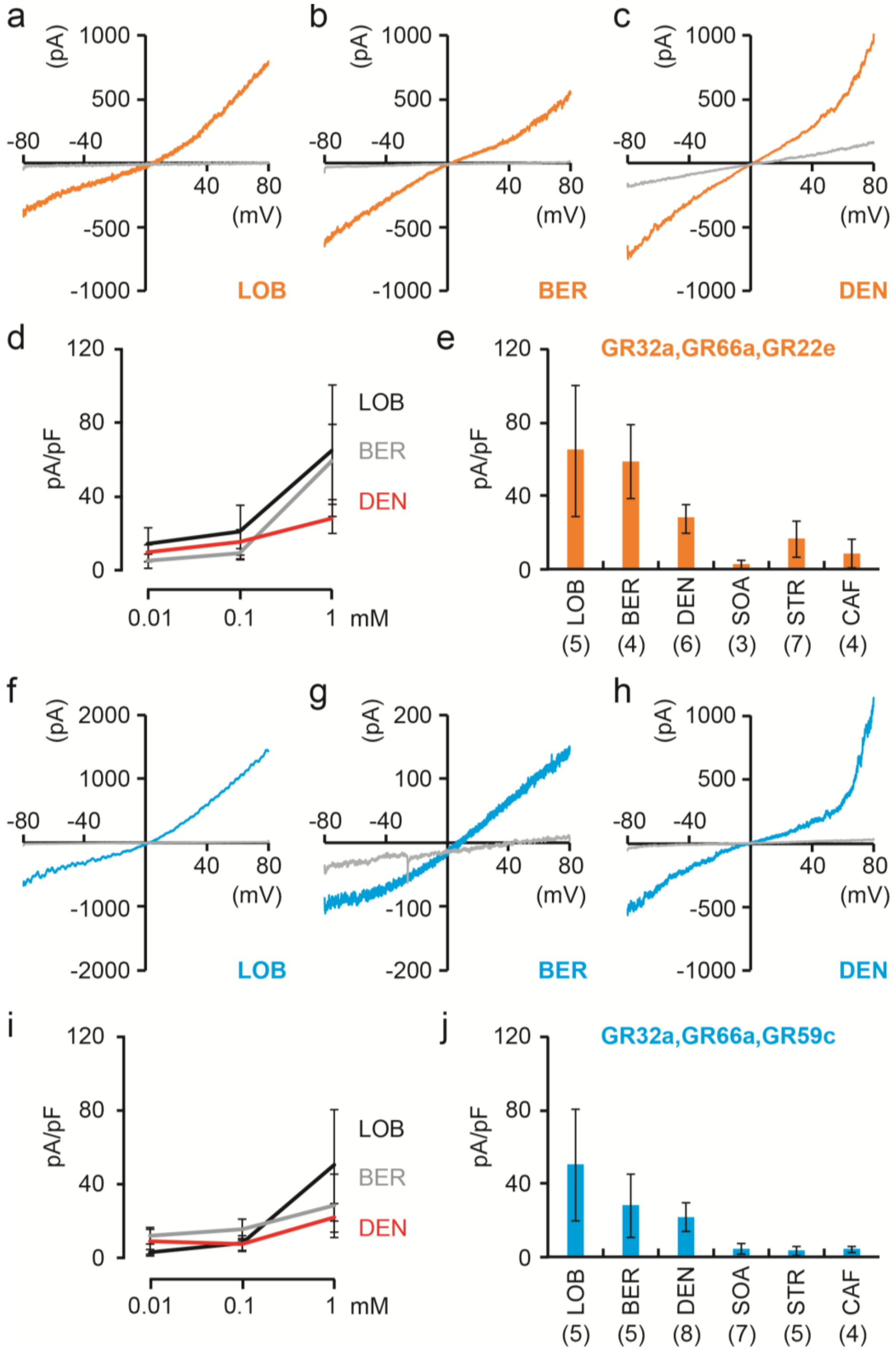

Supplementary Figure 4. Whole-cell voltage clamp recordings of *Gr*-expressing S2 cells.

Cells were stimulated with LOB, BER, or DEN as indicated. All cells were co-transfected

with four plasmids: pActin5c–GAL4, pUAST-EGFP, pUAST-Gr32a, and pUAST-Gr66a. In addition, the cells were also transfected with pUAST-Gr22e (a-e) or pUAST-Gr59c (f-j). (**a**-**c**, **f**-**h**) Representative current-voltage traces produced in response to a 400 ms voltage ramp from −80 mV to +80 mV were obtained in the presence (colored) or absence (gray) of each tastant. (**d**,**i**) Average current at +60 mV in the presence of each tastant at the indicated concentrations (n = 4–6 for each data point). (**e**,**j**) Average current at +60 mV from GR32a, GR66a, and GR22e-expressing cells (e) and from GR32a, GR66a, and GR59c-expressing cells (j) in the presence of 1 mM of each tastant except for 5 mM CAF. The numbers of trials are indicated in the parenthesis. Error bars indicate S.E.M.

Supplementary Table 1

	Genotype	Genes					
		Gr32a	*Gr33a*	*Gr39a.a*	*Gr59c*	*Gr66a*	*Gr89a*
**Total number of transcripts**	*Gr64f-GAL4*	3.8E+09 ±8.9E+08	2.1E+09 ±6.3E+08	2.5E+08 ±4.3E+07	1.8E+08 ±1.9E+07	1.3E+09 ±1.8E+08	2.9E+08 ±2.3E+07
	Gr64f>6 *Gr*s (1 GAL4)	9.7E+09 ±3.7E+08	5.2E+09 ±6.1E+08	1.0E+09 ±3.3E+08	1.8E+09 ±1.1E+08	2.4E+09 ±4.1E+08	6.7E+09 ±3.2E+08
	Gr64f >6 *Gr*s (2 GAL4)	8.0E+09 ±9.4E+08	3.8E+09 ±6.6E+08	1.2E+09 ±3.9E+08	2.2E+09 ±1.7E+08	1.9E+09 ±1.8E+08	8.7E+09 ±1.5E+09
**Number of transcripts in sweet GRNs**	*Gr64f-GAL4*	—	—	—	—	—	—
	Gr64f>6 *Gr*s (1 GAL4)	5.9E+09	3.1E+09	7.8E+08	1.6E+09	1.1E+09	6.4E+09
	2x *Gr64f* >6 *Grs* (2 GAL4)	4.2E+09	1.7E+09	9.2E+08	2.0E+09	5.5E+08	8.4E+09
**Fold change**	*Gr64f-GAL4*	1 ±0.24	1 ±0.30	1 ±0.17	1 ±0.11	1 ±0.14	1 ±0.08
	Gr64f>6 *Gr*s (1 GAL4)	2.56 ±0.10	2.47 ±0.29	4.18 ±1.36	9.81 ±0.61	1.79 ±0.31	22.83 ±1.10
	2x *Gr64f* >6 *Grs* (2 GAL4)	2.10 ±0.25	1.81 ±0.32	4.77 ±1.57	12.26 ±0.92	1.41 ±0.14	29.54 ±4.97

Supplementary Table 1. Expression level of each *Gr* in indicated genotypes. Absolute numbers of transcripts for each *Gr* are estimated from quantitative PCR analysis. The numbers of transcripts are rounded to two decimal places and presented as exponential form. n = 3–5. All data are means ± S.E.M.

Supplementary Table 2

Gr22e	For	GCCACCATGTTCCGGCCTAGTGGAAGTGGCT
	Rev	AAGAATTCTCACAAGTTCCAATAATCGAACTGG
Gr22f	For	ATGAAAATGTTCCAACCTCGTCGC
	Rev	TTACAAGTTCATGAAGTCAAACTGAAGCAGG
Gr32a	For	ATGTCCCCGAACACTTGGGTAATTGAAA
	Rev	TTAAGTTTGTTCTGGTACAGGATCCTCTACTTTC
Gr39a.a	For	GCCACCATGTCAAAAGTCTGCCGGGACCTA
	Rev	TCAAAATTTATTAATAGACTTTGTGGAATTT
Gr39b	For	GCCGCCATGCTCTATTCCTTTCATCC
	Rev	CTAGACTGAACTAAATTGCATAAGCAC
Gr89a	For	GCCGCCATGTTGCGATTTCCGCATGTCTG
	Rev	TTAGATTTCACCATGATTTATATGCTC

Supplementary Table 2. Primer sequences used for cDNA synthesis.

Supplementary Table 3

		5' Homology arm	3' Homology arm
Gr22f^{1}	For	GCGGCCGCAAAGTGCATAGAAAGGTTCGC	GGATCCGTGAGTATATACTGTTGGAATTCGAG
	Rev	GCATGCCTAAAGCCCTGGGTAAAACTAATGTC	GGATCCGGAAACTGCGGTCATCGATGGAC
Gr28a^{1}	For	CGGGGTACCCTCTCATCGAACAGCAGAGTATTGT	AAGGAAAAAAAGCGGCCGCTATAGAGCAATTAAGCGCCTTGGAA
	Rev	CGGGGTACCTGATAAGACCGTTTGATATGGTTTGTG	TCCCCGCGGTTGAACTTTAAATGGTTAACAGGTCGC
Gr36a^{1}	For	GCGGCCGCTTTTTGTGGATCAGCTAAAGCAC	GGATCCATGGTATAGAAGAACTGCACTTGAG
	Rev	GCGGCCGCTTCCGTTAACTGAAATAATTCTC	GGATCCCAGTAACAATTCTAAGTCCGGTC
Gr39b^{1}	For	GGATCCAAATCATATGAGCTCGGGCAAAAC	GCGGCCGCGTTATAAAAAAACAAGAAAGGAAGC
	Rev	GGATCCGCCGTACGGTTGAAAATATACTGGC	GCGGCCGCAACCTAGACGCGGAATTTTTTC
Gr59c^{1}	For	GGATCCGAATAATGTTTTCATTTTCAGCCCGGC	GCGGCCGCTTTCGATTTTCCCCTTCTAC
	Rev	GGTACCGGTTCTCTACTGAAATTGAATTACAGTG	CCGCGGGATAAATCAGGTGGAAACTTTTTC
Gr89a^{1}	For	CGCGGATCCGAATCAAAGGCGATTGTTCTGGTT	AAGGAAAAAAAGCGGCCGCCCAACTATATGCAGTCATTTTGGCT
	Rev	ATTAGGTACCTTTCCGGGCAACTGACCTCCCGGA	TCCCCGCGGCAACTTAAAAAGGCCATAACGGCTTTT

Supplementary Table 3. Primer sequences for the amplification of homology arms for mutant generation.

Supplementary Table 4

	For	Rev
$Gr22f^{1}$	ATGGTTCATGCTGCAGACGACCCT	CCGCTTATTCATGCTTACTCCGAGC
$Gr28a^{1}$	AGCGCTTTTCACAGGCGGACAATG	GGCTATGATTCCGAGCAGCGGATATG
$Gr36a^{1}$	TCGAAATTGACTGGCAAAGAGGTC	CGTTCCATCTCCTTGTGATCATCGA
$Gr39b^{1}$	CGGTTAAAGTGGCAGTCGTATGCT	ACCGTTTCGACACATCCGTTCG
$Gr59c^{1}$	ATGGTTGACTTGGTGAAGACGATTTTGC	CTGCCTCCAGTCGGTTGTCCAATTC
$Gr89a^{1}$	CTTTCAGCACGTGGCCAGTGTCT	ACTATGAAGTTCATCGCATACGAG

Supplementary Table 4. Primer sequences for mutant confirmation.

Supplementary Table 5

Gr32a	For	GGTTTCACCAAACATATTGCAGTGGTGC
	Rev	CAATGATATTCTGATACTCCTTCGACTTGGC
Gr33a	For	GGCAACAAAAGAACGACGATGACGATTTG
	Rev	GGTAAATTGGCTTCCGATGTGTTTCCAG
Gr39a.a	For	CAGCAACCTAGAGGGGTTTCATTCAAG
	Rev	CTTTGCTGTTTTATTTGCCTGATTATGTATGTC
Gr59c	For	CTGCTGATTGTACTATCCACGGCTTCC
	Rev	GCAGTTGGAAGTTCTCAAAGGCTGC
Gr66a	For	CAGAGCACCTTGACTTTCGTGATTGG
	Rev	CCGTCCAATTGTACTGTTGTCCATAACAC
Gr89a	For	CTATGTATCGCTGCATGGAGTTCCCC
	Rev	CATGGCCTGCTCCTGTGTCAATC

Supplementary Table 5. Primer sequences used for quantitative PCR.

Supplementary Table 6

		Abbreviation	Genotypes
Fig. 1	c	*Gr64f*>6 *Grs*	*Gr64f-GAL4,UAS-Gr66a* / *Gr64f-GAL4,UAS-Gr59c* ; *UAS-Gr32a,UAS-Gr33a* / *UAS-Gr39a.a,UAS-Gr89a*
	d	*Gr64f*>6 *Grs*	*Gr64f-GAL4,UAS-Gr66a* / *Gr64f-GAL4,UAS-Gr59c* ; *UAS-Gr32a,UAS-Gr33a* / *UAS-Gr39a.a,UAS-Gr89a*
	e	*Gr64f*>5 *Grs* (-*Gr32a*)	*Gr64f-GAL4,UAS-Gr66a* / *Gr64f-GAL4,UAS-Gr59c* ; *UAS-Gr33a* / *UAS-Gr39a.a,UAS-Gr89a*
		Gr64f>5 *Grs* (-*Gr59c*)	*Gr64f-GAL4,UAS-Gr66a* / *Gr64f-GAL4* ; *UAS-Gr32a,UAS-Gr33a* / *UAS-Gr39a.a,UAS-Gr89a*
		Gr64f>5 *Grs* (-*Gr66a*)	*Gr64f-GAL4* / *Gr64f-GAL4,UAS-Gr59c* ; *UAS-Gr32a,UAS-Gr33a* / *UAS-Gr39a.a,UAS-Gr89a*
		Gr64f>5 *Grs* (-*Gr39a.a*)	*Gr64f-GAL4,UAS-Gr66a* / *Gr64f-GAL4,UAS-Gr59c* ; *UAS-Gr32a,UAS-Gr33a* / *UAS-Gr89a*
		Gr64f>5 *Grs* (-*Gr89a*)	*Gr64f-GAL4,UAS-Gr66a* / *Gr64f-GAL4,UAS-Gr59c* ; *UAS-Gr32a,UAS-Gr33a* / *UAS-Gr39a.a*
	f	*UAS-Gr32a,Gr66a,Gr59c*	*UAS-Gr66a* / *UAS-Gr59c* ; *UAS-Gr32a* / +
		Gr64f>Gr32a,Gr66a,Gr59c	*Gr64f-GAL4,UAS-Gr66a* / *Gr64f-GAL4,UAS-Gr59c* ; *UAS-Gr32a* / +
Fig. 3	f	*UAS-Gr32a,Gr66a,Gr22e*	*UAS-Gr66a* / *UAS-Gr22e* ; *UAS-Gr32a* / +
		Gr64f>Gr32a,Gr66a,Gr22e	*Gr64f-GAL4,UAS-Gr66a* / *Gr64f-GAL4,UAS-Gr22e* ; *UAS-Gr32a* / +
Fig. 4	a b c d	$Gr22e^{1}$;*Gr33a>Gr22e*	$Gr22e^{1}$; *Gr33a-GAL4* / *UAS-Gr22e*
		$Gr22e^{1}$;*Gr33a>Gr59c*	$Gr22e^{1}$; *Gr33a-GAL4* / *UAS-Gr59c*
	e f g h	$Gr59c^{1}$;*Gr33a>Gr22e*	$Gr59c^{1}$; *Gr33a-GAL4* / *UAS-Gr22e*
		$Gr59c^{1}$;*Gr33a>Gr59c*	$Gr59c^{1}$; *Gr33a-GAL4* / *UAS-Gr59c*

		Abbreviation	Genotypes
**Supplementary Fig. 1**	**b**	*Gr64f>Gr32a*	*Gr64f-GAL4* / + ; *UAS-Gr32a* / +
		Gr64f>Gr59c	+ / *Gr64f-GAL4,UAS-Gr59c*
		Gr64f>Gr66a	*Gr64f-GAL4,UAS-Gr66a* / + ;
		Gr64f>Gr32a,Gr59c	*Gr64f-GAL4,UAS-Gr59c* / + ; *UAS-Gr32a* / +
		Gr64f>Gr32a,Gr66a	*Gr64f-GAL4,UAS-Gr66a* / + ; *UAS-Gr32a* / +
		Gr64f>Gr59c, Gr66a	*Gr64f-GAL4,UAS-Gr66a* / *UAS-Gr59c*
	**c**	*Gr64f>Gr32a,Gr66a,Gr59c;+*	*Gr64f-GAL4,UAS-Gr66a* / *Gr64f-GAL4,UAS-Gr59c* ; *UAS-Gr32a* / +
		Gr64f>Gr32a,Gr66a,Gr59c;Gr64af	*Gr64f-GAL4,UAS-Gr66a* / *Gr64f-GAL4,UAS-Gr59c* ; *UAS-Gr32a,Gr64af* / *Gr64af*
**Supplementary Fig. 3**	**a**	*Gr64f>Gr32a,Gr66a,Gr22e;+*	*Gr64f-GAL4,UAS-Gr66a* / *Gr64f-GAL4,UAS-Gr22e* ; *UAS-Gr32a* / +
		Gr64f>Gr32a,Gr66a,Gr22e;Gr64af	*Gr64f-GAL4,UAS-Gr66a* / *Gr64f-GAL4,UAS-Gr22e* ; *UAS-Gr32a,Gr64af* / *Gr64af*
	**b**	*Gr64f>Gr32a,Gr66a,Gr22f*	*Gr64f-GAL4,UAS-Gr66a* / *UAS-Gr22f* ; *UAS-Gr32a* / +
		Gr64f>Gr32a,Gr66a,Gr39b	*Gr64f-GAL4,UAS-Gr66a* / *UAS-Gr39b* ; *UAS-Gr32a* / +

Supplementary Table 6. Complete genotypes of flies in the indicated figures.
